# Atrial Electrography for Postoperative Tachyarrhythmia Analysis in Patients

**DOI:** 10.19102/icrm.2021.121003

**Published:** 2021-10-15

**Authors:** Ashley M. Peotter, Diane R. Brown, Matthew M. Kalscheur, Nicholas H. Von Bergen

**Affiliations:** ^1^The University of Wisconsin School of Medicine and Public Heath, Madison WI, USA; ^2^Division of Pediatric Intensive Care, Department of Pediatrics, University of Wisconsin School of Medicine and Public Heath, Madison WI, USA; ^3^Division of Cardiology, Department of Internal Medicine, University of Wisconsin School of Medicine and Public Heath, Madison WI, USA

**Keywords:** Algorithm, arrhythmia, atrial electrography, electrocardiography, postoperative

## Abstract

The over 400,000 cardiac surgeries performed in the United States each year hold a risk for the postoperative complication of arrhythmias. Currently, bedside monitoring of surface electrocardiogram leads is used to interpret arrhythmias despite the evidence that atrial electrograms (AEGs) offer superior rhythm discrimination. This hesitancy to use the AEG may be due to a lack of training for practitioners in interpreting AEGs; therefore, our goal was to create an algorithm for the diagnosis of tachyarrhythmia using an AEG that can be utilized by any health care practitioner. Our algorithm classifies the most prevalent type of tachyarrhythmias following cardiac surgery. To allow rhythm identification, we categorized them based on their atrial to ventricular signal ratio, which is uniquely apparent on AEGs. Other considerations were given to rhythm regularity, consistency, P-wave axis, and rate. The algorithm includes the most common postoperative arrhythmias differentiated based on a unique branch-point approach, which walks through the steps in arrhythmia discrimination. Both rendered and collected AEGs are included as references for further understanding and interpretation of tachyarrhythmias. The utility of AEGs for rhythm discrimination post-cardiac surgery is established and recent technology can provide real-time and continuous monitoring; however, practitioner training may be inadequate. To bridge this divide, we created an algorithm so that existing atrial wires can be better used for an enhanced rhythm interpretation via AEGs.

## Introduction

Postoperative arrhythmias remain a significant risk for over 400,000 patients who undergo cardiac surgeries in the United States each year.^1^ Atrial fibrillation (AF) alone has a 32% prevalence after coronary artery bypass grafting, and arrhythmias exhibit up to a 64% prevalence after more complex surgeries.^2^ Consequences of postoperative arrhythmia include hemodynamic instability, increased morbidity, length of stay, and cost of care.^3–9^ Given this, careful monitoring, early identification, and appropriate treatment are vital.

**Figure 1:**
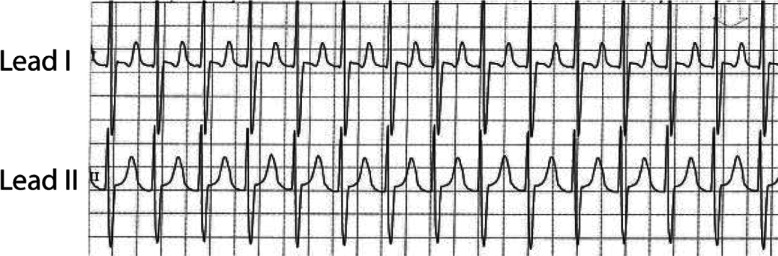
Surface ECGs from the bedside monitor in a postoperative pediatric cardiac patient. The ventricular signals are easily identified, but the P-waves are buried in the T-wave, making their identification challenging.

Most commonly, postoperative arrhythmia monitoring utilizes electrograms from the surface skin leads displayed on the bedside monitor. However, small amplitude atrial signals may make rhythm discrimination difficult or impossible **(Figure 1)**. Due to this challenge, the American Heart Association practice guidelines recommend adapting a 12-lead electrocardiogram (ECG) to visualize atrial signals using temporary atrial epicardial leads for the “improvement of the diagnostic accuracy.”^10^ However, despite evidence of improved diagnostic capabilities, the atrial electrogram (AEG) is not often used in routine postoperative care. This is likely in part due to the lack of timeliness when adapting the 12-lead ECG and in part due to a lack of training for practitioners in AEG interpretation.

**Figure 2:**
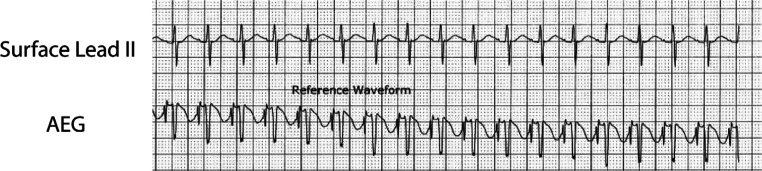
The upper electrogram is from lead II, obtained from the surface leads on the bedside monitor. The lower electrogram is an AEG obtained on the bedside monitor using the AtriAmp. Notice the substantially larger atrial signals on the AEG. AEG: atrial electrogram.

Recently developed technology (AtriAmp; Atrility Medical, Madison, WI, USA) facilitates the transmission of atrial epicardial signal directly from an atrial epicardial wire to the bedside monitor **(Figure 2)**. This advancement can improve the speed and accuracy of diagnosis by providing high-quality and immediately accessible information. This improvement in monitoring reinforces the need for a firm understanding of the AEG interpretation for optimal postoperative management.^11^

Therefore, our goal was to improve the ability of medical providers to accurately identify postoperative tachyarrhythmia using atrial epicardial signals by providing a basic tachycardia flowchart and discussion.

### Understanding the atrial electrogram

**Figure 3:**
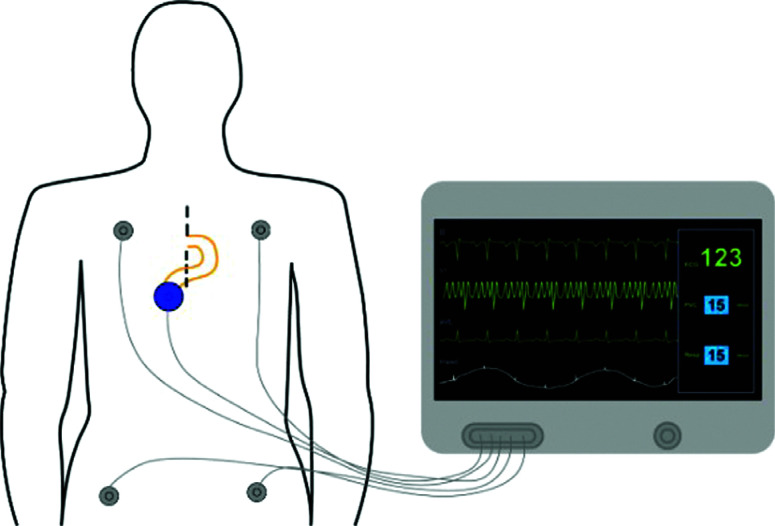
An illustration of how to provide continuous AEG monitoring. The AtriAmp (small blue circle) is attached to the atrial epicardial wires (yellow lines) and the bedside monitor, which displays both the surface ECG and the AEG in real time.

**Figure 4:**
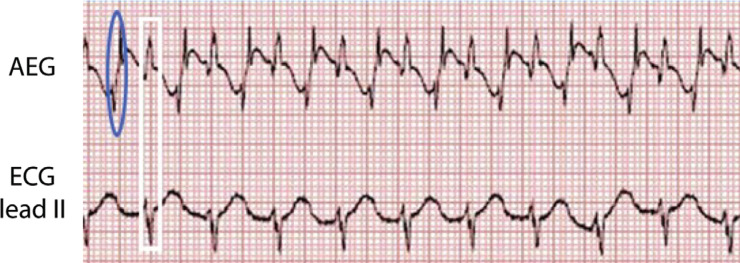
An example of an AEG **(top)** and the surface electrogram **(bottom)**. The atrial signal is circled in blue on the AEG. The ventricular signals are indicated in a white rectangle as seen on the surface lead II and on the AEG. AEG: atrial electrogram; ECG: electrocardiogram.

Adapting the 12-lead ECG by attachment to an atrial epicardial wire, most commonly to the V1 lead, allows a display of the AEG signal on the 12-lead ECG. An AEG can also be obtained using an AtriAmp, which allows continuous monitoring at the bedside as displayed in **Figure 3**. As the timing of the ECG recording is consistent across the vertical axis of the electrograms, it can be simple to determine the atrial and ventricular signals on both the surface electrograms and AEGs as the ventricular signals on the AEGs have similar timing to the QRS complexes on the surface leads **(Figure 4)**. This can allow identification of each ventricular signal on the AEG.

**Figure 5:**
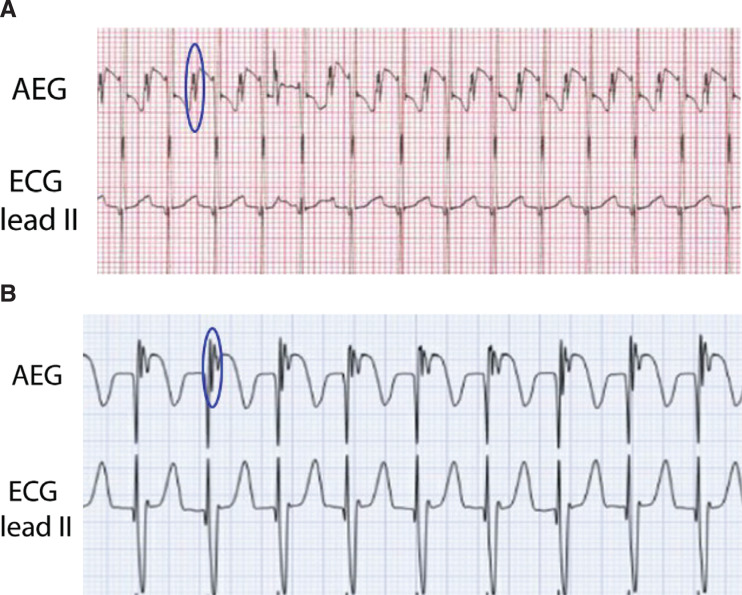
**A:** Top ECG. The AEG and the surface ECG are shown during sinus rhythm. One of the atrial signals is circled in blue. **B:** Another example of an AEG and a surface ECG, this time during a junctional rhythm. Again, one of the atrial signals on the AEG is circled in blue. AEG: atrial electrogram; ECG: electrocardiogram.

A sharp deflection on the AEG, which is absent or smaller on the surface ECG, suggests the atrial signal. The atrial signal may have varying amplitudes in comparison to the ventricular signal. The atrial signal is typically either distinct from the ventricular signal or changes the morphology of the ventricular signal **(Figures 5A and 5B)**. Once the atrial and ventricular signals have been identified, rhythm diagnosis is substantially easier.

Here, we present an algorithm for the diagnosis of tachyarrhythmia using the AEG. We will include a discussion of the arrhythmia with a simplified figure and patient examples. AEGs can be used to distinguish both bradyarrhythmia and tachycardia; however, for this study, we will focus on the evaluation of tachyarrhythmias only.

### Use of the atrial electrogram for rhythm identification

This simple AEG algorithm allows for the evaluation of the atrial and ventricular signals, their relative rates, and their relationship to one another. Our algorithm results in multiple diagnoses in a number of cases. In these cases, we recommend referring to the arrhythmia discussion for further discriminating the details and considering further evaluation as discussed in the additional Maneuvers section.

Though not discussed in detail in this article, it is best to compare an AEG and a surface electrogram concurrently for accurate atrial signal identification. First, identify the ventricular signal on the surface electrogram. This will correspond to the ventricular signal on the AEG **(Figure 4)**. Once the ventricular signals are identified, evaluate the AEG for the atrial signal. These are generally sharp, additional deflections, which may be smaller or absent on the surface ECG.

**Figure 6:**
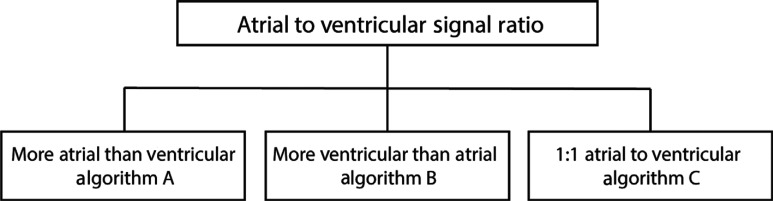
The first step for rhythm identification is to evaluate the A:V ratio.

The next step is to determine the ratio of atrial to ventricular signals **(Figure 6)**. If there are more atrial signals than ventricular signals, the tachyarrhythmia is an atrial-driven rhythm such as AF, intra-atrial reentrant tachycardia (IART) (atrial flutter), atrial ectopic tachycardia (AET) with atrioventricular (AV) conduction block, or sinus tachycardia with AV conduction block. Similarly, if there are more ventricular signals than atrial signals, the rhythm is ventricular, such as ventricular tachycardia (VT) or junctional ectopic tachycardia (JET). Rhythms that have a 1:1 atrial:ventricular (A:V) ratio can be more challenging to diagnose as they can be either atrial, ventricular, or have a component of both.

**Figure 7:**
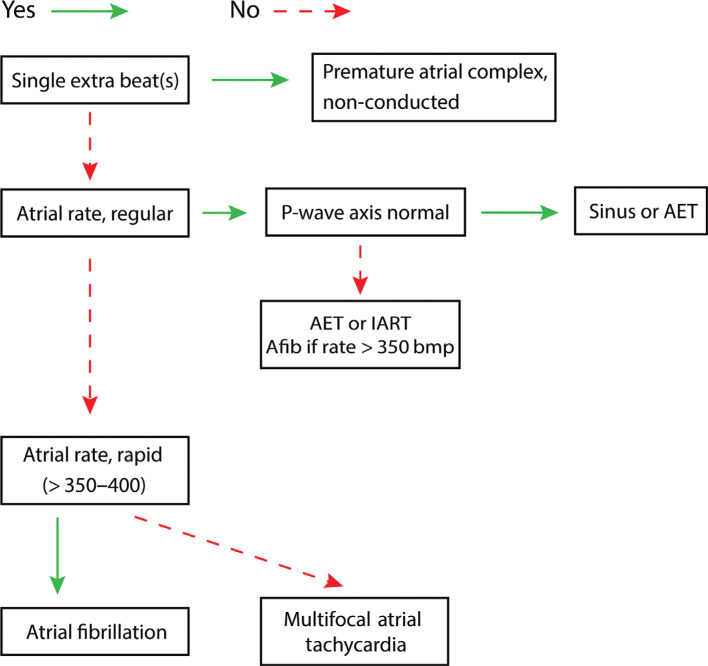
Algorithm A, more atrial than ventricular signals. AET: atrial ectopic tachycardia; Afib: atrial fibrillation; IART: intra-atrial reentrant tachycardia.

***Algorithm A: more atrial signals than ventricular signals (Figure 7).*** The existence of more atrial signals than ventricular signals indicates that a rhythm originating in the atrium is responsible for the tachyarrhythmia. With the exception of pre-excited AF associated with Wolff–Parkinson–White syndrome, the speed of the ventricular response is dependent on the rate of AV nodal conduction. Due to its infrequency, and probable foreknowledge of pre-excitation prior to surgery, pre-excited AF was not included in this algorithm.

To determine the type of atrial arrhythmia, we will evaluate the rate, regularity, and appearance of the atrial signals. In patients with what appears to be a normal P-wave axis suggestive of a sinus origin, the provider must consider AV nodal conduction disease or injury causing some degree of heart block when associated with more atrial than ventricular signals. This is not especially uncommon in patients after cardiac surgery.

**Figure 8:**
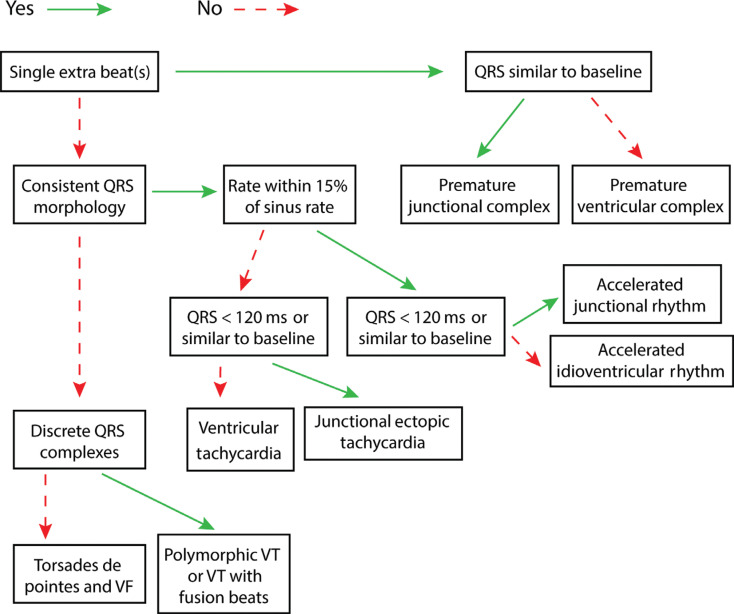
Algorithm B, more ventricular than atrial signals. VF: ventricular fibrillation; VT: ventricular tachycardia.

***Algorithm B: more ventricular signals than atrial signals (Figure 8).*** The confident assertion that there are more ventricular signals than atrial signals is often the most difficult step to confirm the presence of ventricular arrhythmias. Without this confirmation, arrhythmias in the presence of pre-existing bundle branch block or with aberrancy can be difficult to exclude. To best classify these arrhythmias, we need to evaluate the rate, the regularity, and the appearance of the QRS complex. Again, a more detailed discussion of the individual arrhythmias and their AEG follows the algorithms.

**Figure 9:**
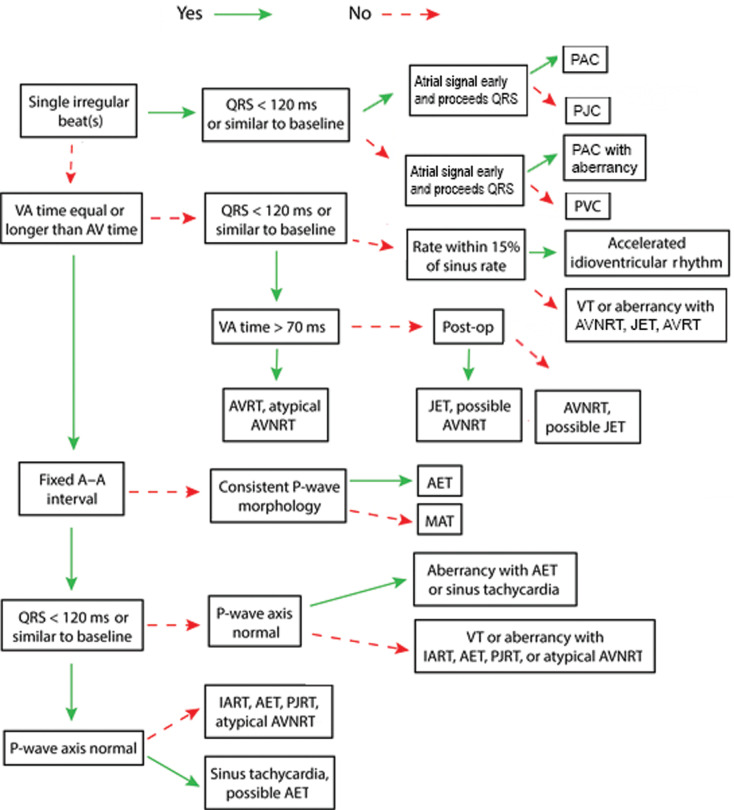
Algorithm C, 1:1 atrial to ventricular signal ratio. A–A: atrial to atrial; A–V: time from the atrial signals to the ventricular signals; AET: atrial ectopic tachycardia; AV: atrioventricular; AVNRT: atrioventricular nodal reentrant tachycardia; AVRT: atrioventricular reentrant tachycardia; IART: intra-atrial reentrant tachycardia; JET: junctional ectopic tachycardia; MAT: multifocal atrial tachycardia; PAC: premature atrial complex; PJC: premature junctional complex; PJRT: permanent junctional reciprocating tachycardia; PVC: premature ventricular complex; V–A: time from the ventricular signals to the atrial signals; VT: ventricular tachycardia.

***Algorithm C: 1:1 atrial to ventricular signal ratio (Figure 9).*** When arrhythmias have a 1:1 A:V ratio, the rhythm could originate from either the atria or the ventricle or could have a component of a reentrant loop within both chambers. If the clinical situation allows, adenosine can help uncover the origin of arrhythmias. If adenosine results in more atrial or more ventricular signals, this implies both that the AV node was not within a reentrant circuit and indicates the origin of the arrhythmia from the chamber with the greater number of signals. In these cases, where the A:V ratio after adenosine is not 1:1, algorithm A or B can be used to diagnose the arrhythmia as appropriate. As many of the common supraventricular tachycardias are AV node–dependent, adenosine may convert the rhythm to sinus rhythm. This suggests that the arrhythmia utilizes the AV node, though some focal arrhythmias (both atrial and ventricular) can be adenosine-sensitive.

**Figure 10:**
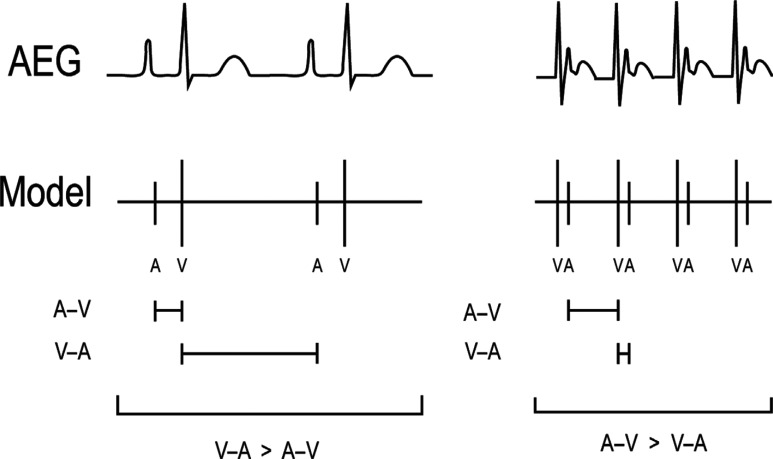
A method to compare the A–V time and the V–A time. The atrial signal is labeled A, while the ventricular signal is labeled V. The V–A time is longer than the A–V time on the left example, and the A–V time is longer than the V–A time on the right example. A–V: time from the atrial signals to the ventricular signals; AEG: atrial electrogram; V–A: the time from the ventricular signals to the atrial signals.

With a 1:1 A:V ratio, it may be required to evaluate the timing between the atrial and ventricular signals, the regularity, and the appearance of the QRS complex. The first step will be to compare the time from the atrial signals to the ventricular signals (A–V time) in comparison to the time from the ventricular signals to the atrial signals (V–A time) as shown in **Figure 10**.

### Maneuvers **(Figure 11)**

As the AEG is obtained from the temporary atrial epicardial wires, atrial pacing should be considered as atrial pacing maneuvers can assist with treatment and identification of the tachyarrhythmia. Many of the reentrant rhythms [IART, atrioventricular nodal reentrant tachycardia (AVNRT), atrioventricular reentrant tachycardia (AVRT)] can convert to sinus with atrial overdrive pacing. Many of the automatic rhythms (AET, JET, idioventricular rhythms) can be suppressed during pacing at a higher rate than the arrhythmia. However, unlike the re-entrant rhythms, automatic rhythms often resurface after the atrial pacing is stopped. Though more complex measurements can be performed after atrial overdrive pacing to help determine the arrhythmia diagnosis, this is beyond the scope of this article.

Adenosine can also be used to assist with identification and treatment. Adenosine blocks AV nodal conduction resulting in the termination of AV node–dependent arrhythmias [AVRT, AVNRT, atypical AVNRT, permanent junctional reciprocating tachycardia (PJRT)]. It can also terminate some automatic rhythms, such as some AETs and some VTs. As discussed already, in an arrhythmia with 1:1 conduction, if adenosine blocks AV nodal conduction and the arrhythmia is not terminated, the resulting arrhythmia (with more atrial than ventricular or more ventricular than atrial signals) can be used to diagnose the arrhythmia.

## Arrhythmias and their atrial electrograms

In this section, we discuss the individual appearance of the AEG in each arrhythmia type and their typical rate, behavior, and response to adenosine and/or atrial overdrive pacing. We include both an idealized figure of the AEG during the arrhythmia as well as patient examples when available. Unless specified otherwise, the ECGs are displayed with a standard sweep speed of 25 mm/s.

### Accelerated junctional rhythm **(Figure 12)**

***Background.*** An accelerated junctional rhythm in the postoperative setting may be a precursor to the more rapid JET and has similar findings to JET with the exception of a slower rate. An accelerated junctional rhythm is generally within 15% of the sinus rhythm.

**Figure 12:**
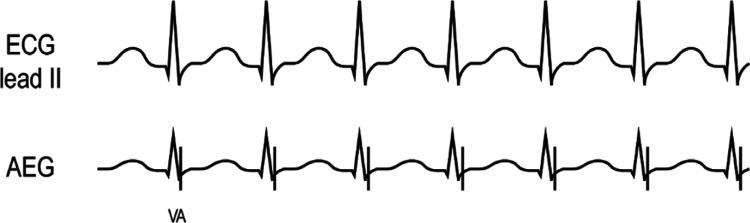
Accelerated junctional rhythm illustration. AEG: atrial electrogram; ECG: electrocardiogram.

***Atrial electrogram.*** An accelerated junctional rhythm may show 1:1 atrial to ventricular signals or more ventricular signals depending on the retrograde AV nodal conduction **(Figure 12)**. In comparison to a slow AVNRT, which may appear similar on the AEG, it is more common postoperatively, has more rate variability, and most often has a gradual onset. During accelerated junctional rhythm, the atrial signals often closely follow the ventricular signals, either on or near the terminal QRS complex. This can appear very similar to the electrogram seen with junctional ectopic tachycardia (JET) (refer to **Figure 19B**, later), though the rates during an accelerated junctional rhythm are slower. When rates are close to the sinus rhythm, an accelerated junctional rhythm may intersperse with the sinus rhythm.

***Maneuvers.*** An accelerated junctional rhythm is generally not responsive to adenosine. Atrial pacing at a rate higher than the accelerated junctional rhythm suppresses the arrhythmia until pacing is discontinued.

### Accelerated ventricular rhythm **(Figures 13A and 13B)**

***Background.*** An accelerated ventricular rhythm reveals a regular, wide complex rhythm with a relatively slow rate, typically within 15% of the sinus rate. This is better tolerated than the more rapid VT.

**Figure 13:**
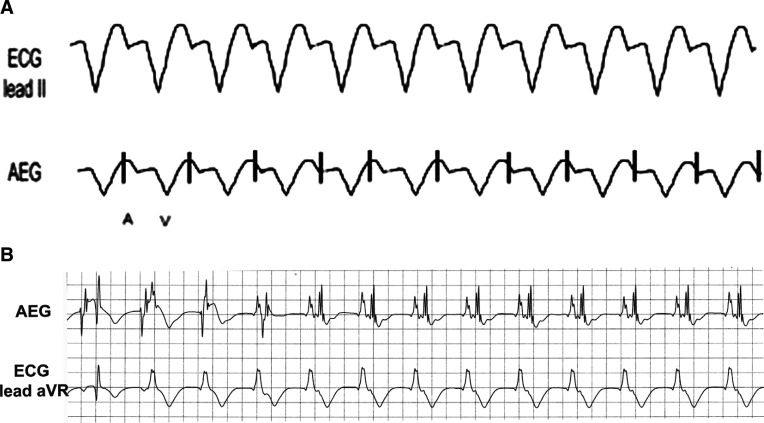
**A:** Accelerated ventricular rhythm illustration. **B:** This patient example starts as a sinus rhythm followed by an accelerated ventricular rhythm with retrograde atrial activation. AEG: atrial electrogram; ECG: electrocardiogram.

***Atrial electrogram.*** The A:V ratio is dependent on the AV nodal conduction and can show either a 1:1 A:V ratio or more ventricular than atrial signals. In comparison to an accelerated junctional rhythm, an accelerated ventricular rhythm has a morphology that is wide complex and distinct from the patient’s baseline QRS appearance. **Figure 13A** shows an accelerated ventricular rhythm with intact retrograde conduction and a 1:1 A:V ratio. **Figure 13B** shows the ECG and AEG of a postoperative child, initially with a sinus beat for comparison to the following idioventricular rhythm with intact retrograde conduction. Note the wide complex rhythm and the large atrial signal following the QRS on the AEG.

***Maneuvers.*** An accelerated ventricular rhythm is generally not responsive to adenosine. Atrial pacing at a rate higher than the accelerated ventricular rhythm suppresses the arrhythmia until pacing is discontinued.

### Atrial ectopic tachycardia (or atrial tachycardia) **(Figures 14A and 14B)**

***Background.*** AET, sometimes called atrial tachycardia, is a somewhat rare arrhythmia postoperatively.

**Figure 14:**
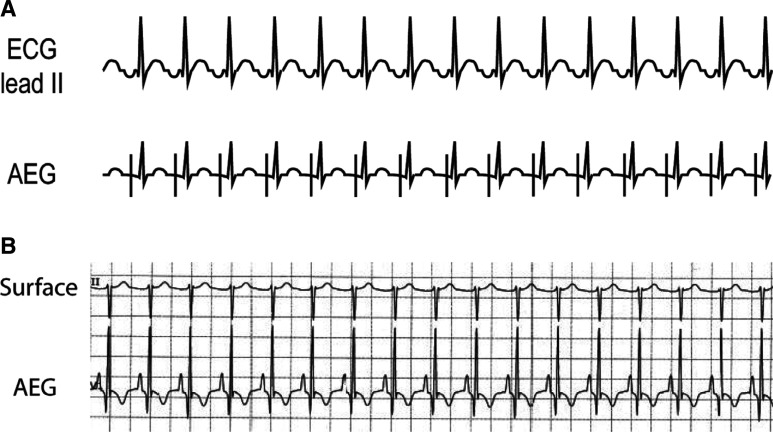
**A:** Illustration of AET. **B:** Patient example of AET. AEG: atrial electrogram; ECG: electrocardiogram.

***Atrial electrogram.*** Depending on the rate and the AV nodal conduction, there can be either a 1:1 A:V ratio or a greater number of atrial signals. **Figure 14A** shows a simplified AET example with a 1:1 A:V rhythm. **Figure 14B** shows the surface ECG and AEG of a child after cardiac surgery. In **Figure 14B**, the P-wave is difficult to visualize in lead II, but the atrial signals were easily seen on the AEG. In some cases, especially after congenital heart disease surgery, AET may present with variable AV conduction if there are rapid rates or AV conduction disease. The appearance of the P-wave can provide insight into the source of the arrhythmia.

***Maneuvers.*** Though AET is generally not converted with adenosine, it can occasionally slow down or terminate; therefore, the response to adenosine is nondiagnostic. AET can be suppressed with overdrive pacing, though the AET often resumes once pacing is stopped.

### Atrial fibrillation **(Figures 15A and 15B)**

***Background.*** AF is one of the most common postoperative arrhythmias in the non-pediatric population.

**Figure 15:**
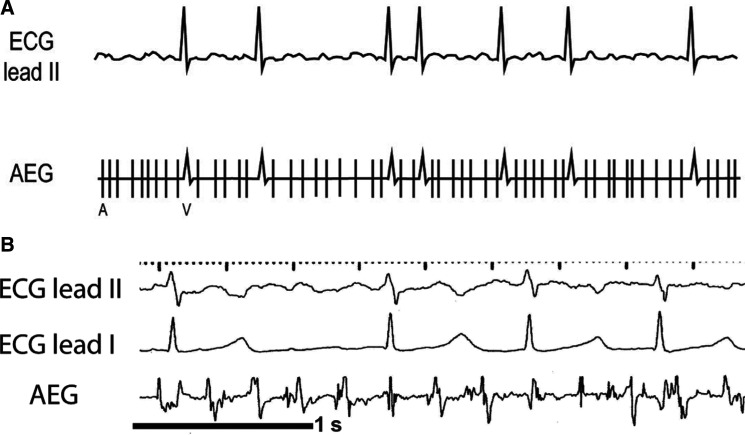
**A:** Illustration of AF. **B:** Patient example of AF. AEG: atrial electrogram; ECG: electrocardiogram.

***Atrial electrogram.*** AF typically shows rapid (> 350 bpm) atrial signals, with an irregular, slower ventricular rate. As with other atrial arrhythmias, the ventricular rate is dependent on the rate of conduction through the AV node. Though the atrial signals generally appear irregular on the surface electrogram, they may appear more uniform on an AEG. The patient example in **Figure 15B** shows a relatively coarse AF, but even so, notice the small electrograms between the larger atrial spikes, which can also help differentiate from IART.

***Maneuvers.*** AF is not responsive to adenosine though the resulting AV block can make AF more easily visible. AF is not responsive to atrial overdrive pacing.

### Atrioventricular nodal reentrant tachycardia **(Figures 16A and 16B)**

***Background.*** Though AVNRT is the most common paroxysmal supraventricular tachycardia in older children and young adults, it is relatively uncommon after heart surgery. It appears very similar to JET; though as an AV node–dependent reentrant rhythm, it generally displays a sudden onset and termination with a consistent and rapid rate.

**Figure 16:**
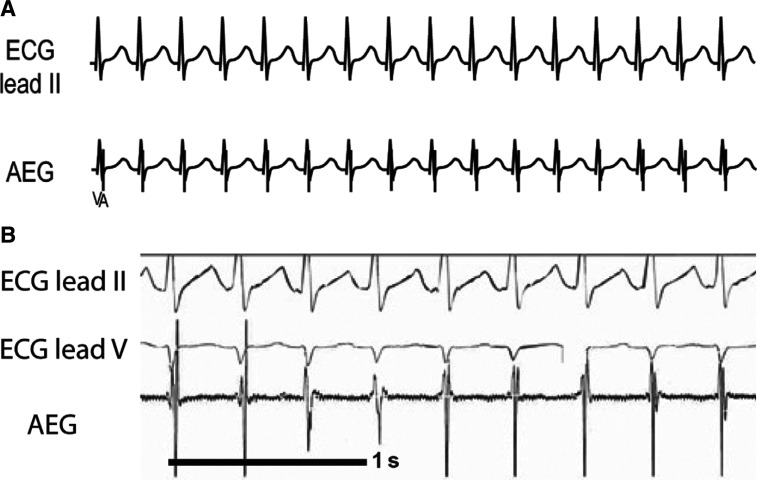
**A:** Illustration of AVNRT. **B:** Patient example of AVNRT. AEG: atrial electrogram; ECG: electrocardiogram.

***Atrial electrogram.*** AVNRT shows a 1:1 A:V ratio, with an atrial signal near or overlapping the terminal portion of the QRS complex with a V–A time typically less than 70 ms. The AEG appears similar to an AEG of JET, though AVNRT is less common than JET after heart surgery. AVNRT is more likely to involve a higher heart rate (often > 220 bpm in children and young adults) and demonstrate a sudden onset than JET. This patient example **(Figure 16B)**, taken from an intracardiac AEG, shows an atrial signal overlapping the terminal QRS complex.

***Maneuvers.*** AVNRT can be converted to sinus with adenosine. AVNRT can be converted to sinus with atrial overdrive pacing.

### Atrioventricular reentrant tachycardia **(Figures 17A and 17B)**

***Background.*** Though AVRT is common in an outpatient setting, it is uncommon in the postoperative setting. It is a re-entrant rhythm, so it generally exhibits a sudden onset and sudden termination with rates in excess of 200 bpm. AVRT is AV node–dependent.

**Figure 17:**
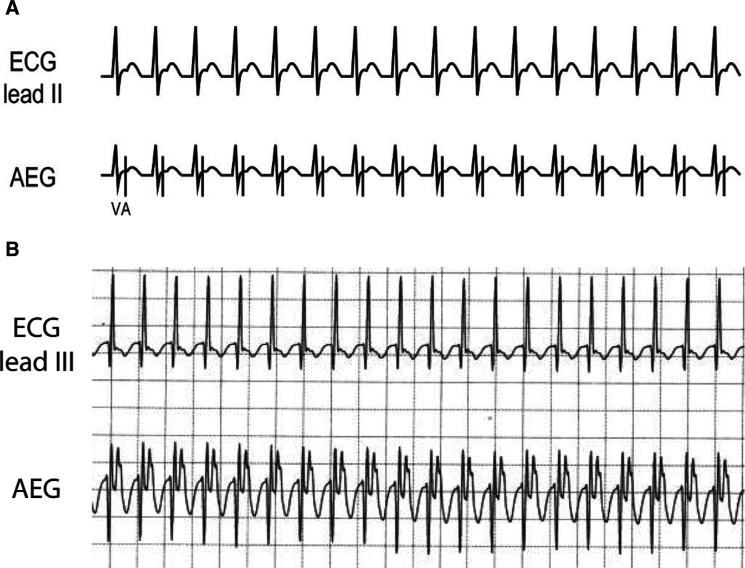
**A:** Illustration of AVRT. **B:** Patient example of AVRT. AEG: atrial electrogram; ECG: electrocardiogram.

***Atrial electrogram.*** Orthodromic AVRT demonstrates a 1:1 A:V ratio with the atrial signal typically located just following the QRS complex in the early T-wave. Unlike AVNRT (or JET), the atrial signal is generally more than 70 ms from the QRS complex. In the patient example **(Figure 17B)**, though the P-wave is difficult to distinguish on the surface lead, the atrial signal on the AEG is easily seen following the QRS complex in the early T-wave. This infant had a sudden increase in heart rate and was successfully atrial overdrive–paced.

***Maneuvers.*** As AVRT is AV node–dependent, it can be converted to sinus with adenosine. AVRT can be converted to sinus with atrial overdrive pacing.

### Intra-atrial reentrant tachycardia **(Figures 18A and 18B)**

***Background.*** IART is not unusual after some of the more complex heart surgeries due to the potential for more extensive atrial scar providing additional arrhythmia substrate.

**Figure 18:**
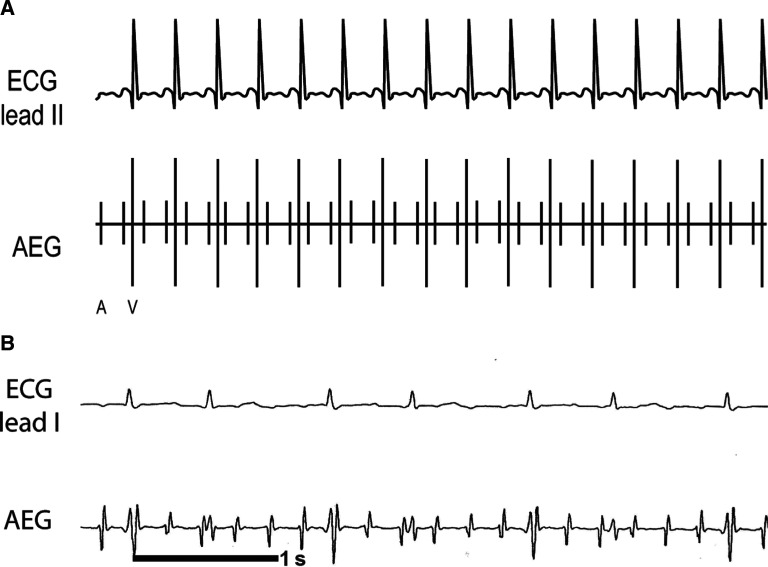
**A:** Illustration of IART. **B:** Patient example of IART. AEG: atrial electrogram; ECG: electrocardiogram.

***Atrial electrogram.*** Typically, IART has a rapid fixed atrial rate. Atrial rates are often between 200 and 350 bpm, though rates may be slower in patients with coronary heart disease (CHD) or diseased myocardium. Depending on the AV nodal conduction, IART may show regular A–V and V–V timing, such as seen with 2:1 or 3:1 AV conduction. Alternatively, the AEG may also show an irregular ventricular rate if there is inconsistent AV nodal conduction. **Figure 18A** demonstrates IART with a 2:1 AV conduction. The patient electrogram **(Figure 18B)** shows a consistent IART cycle length (~300 ms) with variable AV nodal conduction. In comparison to the AF AEG, IART shows less variability in the rate of the AEG and less variability in the size of the AEG signals.

***Maneuvers.*** IART is not adenosine-sensitive. Adenosine can be used to more easily view the atrial complexes by blocking the AV nodal conduction and ventricular signals. IART may be converted to sinus with atrial overdrive pacing.

### Junctional ectopic tachycardia **(Figures 19A and 19B)**

***Background.*** JET is most commonly found in the postoperative setting. It has a similar QRS appearance to the baseline rhythm and most often occurs within 48 to 72 hours of surgery.

**Figure 19:**
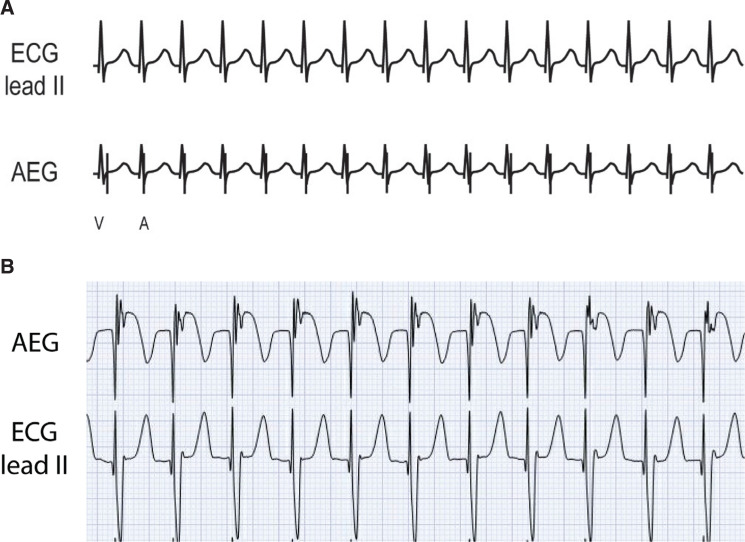
**A:** Illustration of JET. **B:** Patient example of JET. AEG: atrial electrogram; ECG: electrocardiogram.

***Atrial electrogram.*** JET may show a 1:1 AV conduction or more ventricular than atrial signals. The AEG generally first has a ventricular signal, and appears with intact retrograde conduction, with an atrial signal closely following or overlapping the terminal QRS complex. The AEG often appears very similar to AVNRT. However, JET generally shows rate variability and gradual onset. The patient example **(Figure 19B)** shows the atrial signals on the terminal end of the QRS complex.

***Maneuvers.*** JET is generally not responsive to adenosine. Atrial pacing at a rate higher than the JET may suppress the arrhythmia. However, unless controlled by other means, the rhythm typically returns when pacing is discontinued.

### Multifocal atrial tachycardia **(Figure 20)**

***Background.*** Multifocal atrial tachycardia (MAT) is uncommon postoperatively and appears to be more frequent in patients with concurrent pulmonary concerns.

**Figure 20:**
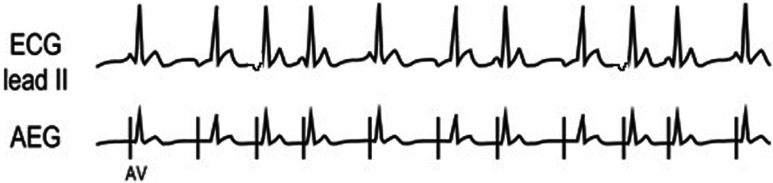
Illustration of MAT. AEG: atrial electrogram; ECG: electrocardiogram.

***Atrial electrogram.*** MAT typically has an irregular atrial rate, an irregular ventricular rate, and a variable P-wave morphology, often interspersed with periods of sinus rhythm **(Figure 20)**. This arrhythmia is due to multiple sites of automaticity within the atrium resulting in variable atrial signals and P-wave morphology. It may demonstrate both a 1:1 A:V ratio and more atrial than ventricular signals.

***Maneuvers.*** MAT is typically not responsive to adenosine. MAT may be suppressed with overdrive pacing, though it typically returns after pacing has stopped.

### Persistent junctional reciprocating tachycardia **(Figure 21)**

***Background.*** PJRT is an uncommon arrhythmia in an outpatient setting, and is even less common in the postoperative setting. On the surface electrogram, it is characterized by P-waves originating from the lower right atrium (RA). The rhythm is often relatively slow and “persistent.”

**Figure 21:**
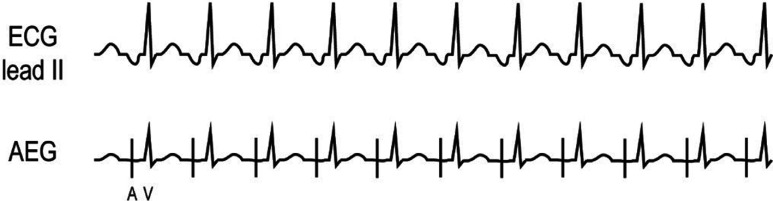
Illustration of PJRT. AEG: atrial electrogram; ECG: electrocardiogram.

***Atrial electrogram.*** PJRT has a 1:1 A:V conduction with a V–A time longer than the A–V time **(Figure 21)**. It is often somewhat slower and more incessant than other re-entrant arrhythmias. The P-wave originates from the lower RA. In comparison to AET, PJRT typically shows a sudden onset and sudden termination, with less rate variability. It can appear similar to a slow IART, which is occasionally seen in patients with CHD.

***Maneuvers.*** PJRT can be converted to sinus with adenosine. PJRT can be converted to sinus with atrial overdrive pacing.

### Premature atrial complex **(Figures 22A and 22B)**

***Background.*** Premature atrial complexes (PACs) are common after heart surgery. Often, they do not require significant intervention unless resulting in unfavorable hemodynamics.

**Figure 22:**
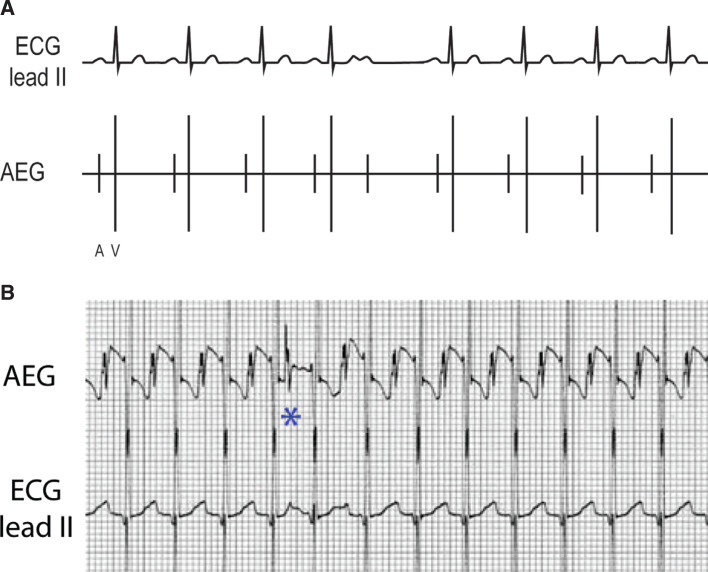
**A:** Illustration of PAC. **B:** Patient example of PAC. The fifth atrial beat is marked with an asterisk. AEG: atrial electrogram; ECG: electrocardiogram.

***Atrial electrogram.*** The AEG shows an early atrial signal. If a PAC reaches the AV node while the node is still refractory, it will result in an early atrial complex without a following ventricular signal, termed a blocked PAC. If the PACs occur after AV nodal repolarization, it will result in an early atrial and early ventricular complex. **Figure 22A** shows a blocked PAC as represented by the early atrial signal with no subsequent ventricular signal. The patient example **(Figure 22B)** demonstrates a PAC (the fifth atrial beat, marked with an asterisk) with intact AV conduction resulting in an earlier atrial and ventricular signal.

***Maneuvers.*** PACs are not typically responsive to adenosine. Overdrive pacing can occasionally suppress PACs, which typically recur after pacing has stopped.

### Premature junctional complex **(Figure 23)**

***Background.*** Premature junctional complexes (PJCs) are due to an early junctional beat originating from the AV nodal area, resulting in an early ventricular complex with a similar appearance to the baseline rhythm. They are relatively uncommon after heart surgery.

**Figure 23:**
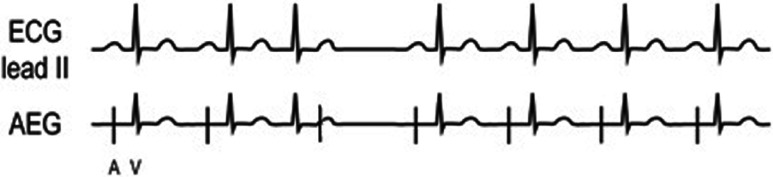
Illustration of PJC. AEG: atrial electrogram; ECG: electrocardiogram.

***Atrial electrogram.*** PJCs have a similar appearance to the baseline QRS complex but are single and early without a proceeding atrial signal **(Figure 23)**. Similar to PVCs, the A:V ratio in PJCs may demonstrate more ventricular than atrial signals or a 1:1 A:V ratio. A PJC may or may not result in retrograde conduction, which could alter the atrial cycle length.

***Maneuvers.*** Adenosine is not effective for the treatment or diagnosis of PJCs. Though atrial overdrive pacing is not recommended for the treatment of PJCs, PJCs may be less common when the ventricular rate is higher.

### Premature ventricular complex **(Figures 24A and 24B)**

***Background***. Premature ventricular complexes (PVCs) are seen frequently after heart surgery. They are due to an early impulse from the ventricle, which may or may not have retrograde conduction through the AV node.

**Figure 24:**
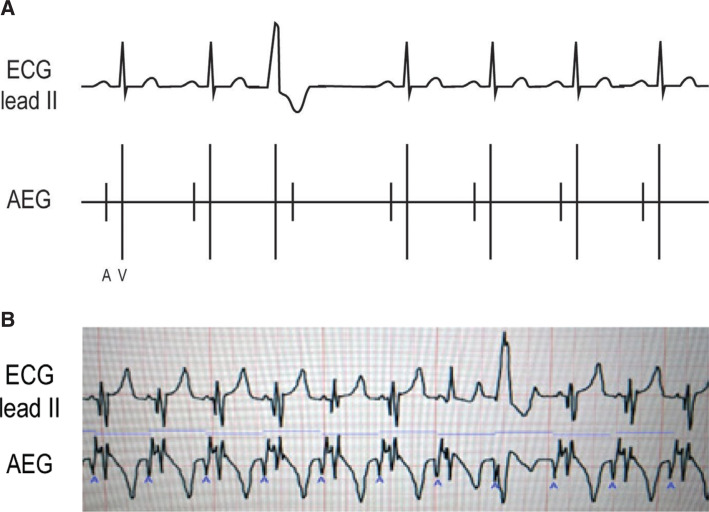
**A:** Illustration of PVC. **B:** Patient example of PVC. AEG: atrial electrogram; ECG: electrocardiogram.

***Atrial electrogram.*** Most commonly, the A:V ratio in PVCs demonstrates a 1:1 A:V ratio; however, in some cases, there may be more ventricular than atrial signals depending on the PVC timing and retrograde AV nodal conduction. PVCs show an early ventricular signal with a different appearance than the baseline QRS complex. Frequently, a PVC may occur that does not depolarize the atria and results in no alteration in the timing of the atrial signal. The patient example **(Figure 24B)** demonstrates this behavior. In this example, we have labeled the atrial signals for clarity (small blue A) and marked out a consistent atrial to atrial duration with a small blue bar. Notice that there is an early, wide complex ventricular signal with no alteration in the atrial cycle length.

***Maneuvers.*** Adenosine is not effective for the treatment or diagnosis of PVCs. Though atrial overdrive pacing is not recommended for the treatment of PVCs, PVCs may be less common when the ventricular rate is higher.

### Sinus tachycardia **(Figures 25A–25D)**

***Background.*** Sinus tachycardia is extremely common after heart surgery and may be due to varied causes such as pain, fever, poor cardiac output, hypovolemia, medications, and more. The atrial rate during sinus rhythm may be responsive to treatment of these underlying concerns, which can assist in the diagnosis of the tachycardia.

**Figure 25:**
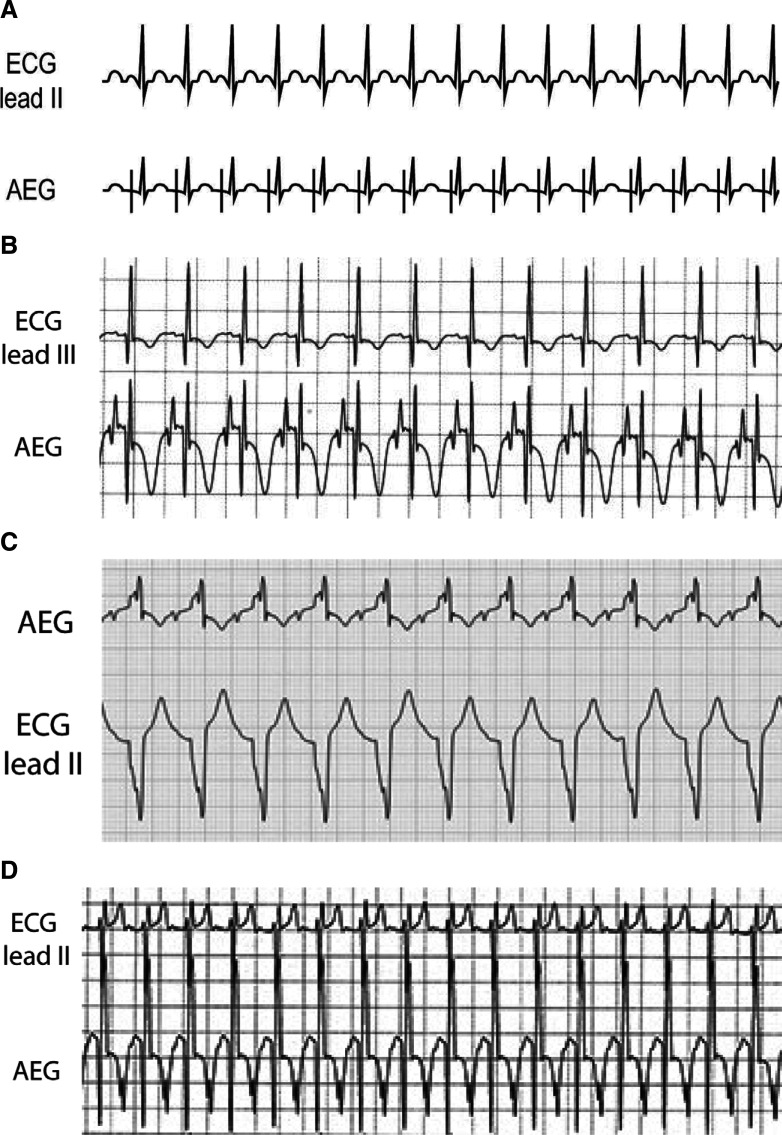
**A:** Illustration of sinus tachycardia. **B–D:** Patient examples of sinus tachycardia. AEG: atrial electrogram; ECG: electrocardiogram.

***Atrial electrogram.*** Sinus tachycardia generally has atrial rates less than 200 bpm, though, especially in infants, rates can occasionally approach (or pass) 210 bpm. Sinus tachycardia generally has a gradual increase and decrease in rate, which can help distinguish it from many re-entrant arrhythmias. It has a normal P-wave axis, though this can be difficult to distinguish on the surface leads. Generally, the AEG demonstrates a 1:1 A:V ratio with a longer V–A time than A–V time. We have included multiple patient examples due to the frequency of this arrhythmia and the importance of distinguishing it from arrhythmias such as JET. The first patient example **(Figure 25B)** represents sinus tachycardia, with the prominent atrial signals seen on the AEG and much smaller atrial signals on lead III. **Figure 25C** demonstrates sinus tachycardia in a patient with an underlying right bundle branch block. Note the somewhat smaller AEG atrial signal following the T-wave (not seen on the surface lead). **Figure 25D** demonstrates a large atrial signal on the AEG just after the peak of the T-wave. This can be seen as an inverted deflection in the T-wave in lead III.

***Maneuvers.*** Sinus tachycardia will often transiently slow down after adenosine. Sinus rhythm will be suppressed when pacing a rate higher than that of the sinus tachycardia. The rhythm will resume once pacing has stopped.

### Ventricular tachycardia **(Figures 26A–26C)**

***Background.*** VT is not uncommon after cardiac surgery, especially when non-sustained. However, given the potential for hemodynamic compromise, it warrants careful evaluation.

**Figure 26:**
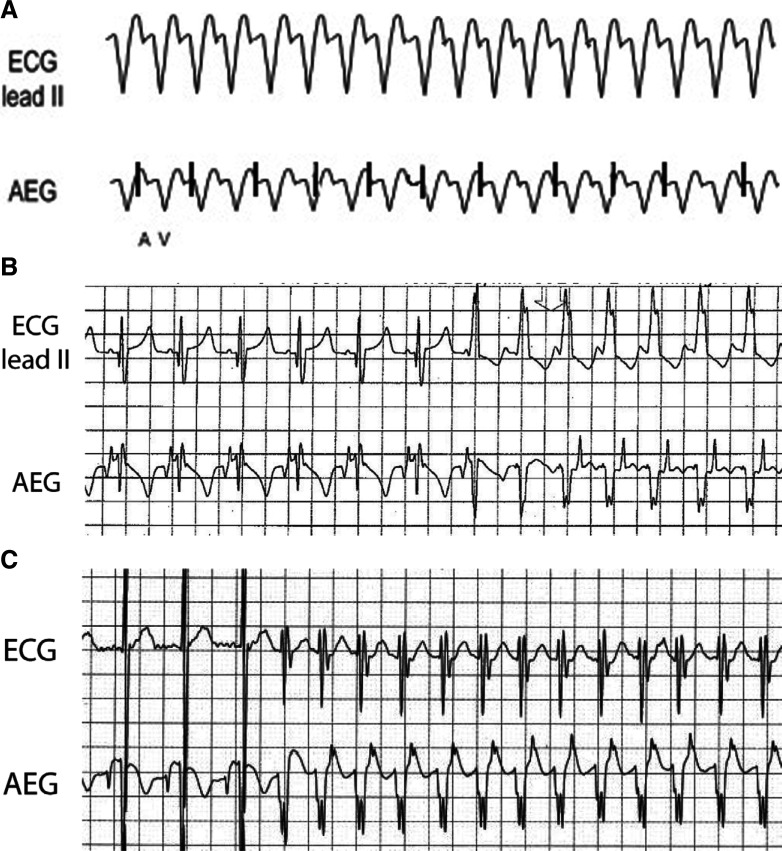
**A:** Illustration of VT. **B and C:** Patient examples of VT. AEG: atrial electrogram; ECG: electrocardiogram.

***Atrial electrogram.*** VT reveals a regular rhythm with a wide QRS complex distinct from the baseline QRS appearance. The A:V ratio is dependent on the retrograde AV nodal conduction as VT can show both a 1:1 A:V ratio and more ventricular than atrial signals. In comparison to JET, VT has a morphology that is wide and distinct from the patient’s baseline QRS appearance. Evaluating the transition in/out of the arrhythmia can be especially helpful for diagnosis. **Figure 26A** shows a wide complex rhythm with more ventricular than atrial signals. In **Figure 26B**, the ECG and AEG of a postoperative pediatric patient with a run of VT with retrograde conduction are shown. We included the onset of the arrhythmia for clarity. Note the sinus rhythm followed by a wide complex rhythm. The ventricular signals accelerate ahead of the atrial signals, which can be identified as the large upward sharp deflection on the early T-wave. A similar finding is seen on **Figure 26C**, again with sinus followed by an early ventricular beat with no proceeding atrial signal, eventually followed by (in this case) smaller amplitude atrial signals on the T-wave.

***Maneuvers.*** Though there are exceptions, VT is generally not responsive to adenosine or to atrial overdrive pacing.

### Ventricular tachycardia (polymorphic)/torsades de pointes/ventricular fibrillation **(Figures 27A and 27B)**

***Background.*** We have included these three arrhythmias together despite slight differences between them. Polymorphic VT is characterized by different morphologies of a wide QRS complex tachycardia. Torsades de pointes represents a characteristic appearance of rotating ventricular wavefront, and ventricular fibrillation (VF) shows rapid ventricular fibrillatory waves with no discrete QRS complexes. These rhythms are hemodynamically unstable and should be assessed and treated immediately.

**Figure 27:**
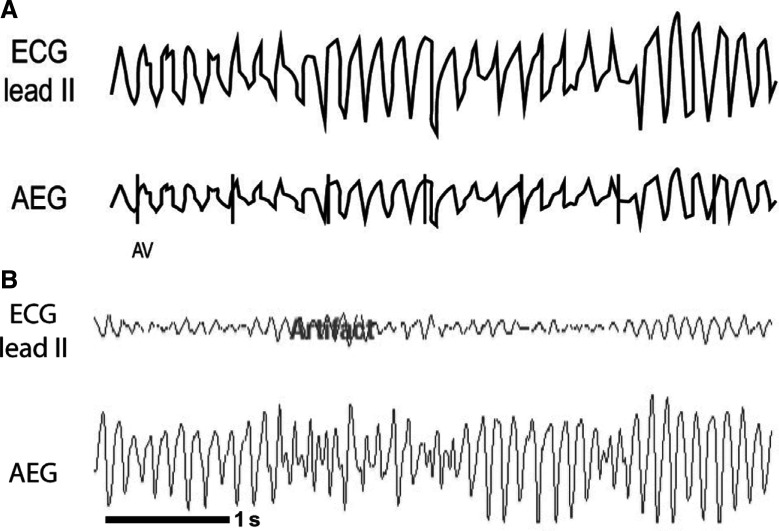
**A:** Illustration of VT (polymorphic)/torsades de pointes/VF. **B:** Animal model example of VF. AEG: atrial electrogram; ECG: electrocardiogram.

***Atrial electrogram.*** Rapid ventricular signals with varying morphologies are seen. The atrial signals may be substantially slower or non-existent. The QRS morphology will be less consistent and less regular than typical VT. In this case, the “patient” example **(Figure 27B)** is from an animal model, but represents the findings in humans as well. It shows the VF waves, but does not demonstrate discrete atrial signals. In some cases, the atrial signals can be more prominent, as seen in **Figure 27A**. If prominent atrial signals are seen without underlying ventricular signals, they may be mistaken for a QRS complex (and the rhythm mistaken for AF).

***Maneuvers.*** Polymorphic VT/torsades/VF is not responsive to adenosine. Defibrillation may be required. Atrial overdrive pacing is not effective. Defibrillation may be required.

## Discussion

Abnormal heart rhythms are common after cardiac surgery, and prompt identification and treatment can be vital in these potentially critical patients. Unfortunately, standard surface electrogram monitoring is often inadequate to accurately diagnose arrhythmias, resulting in delayed diagnosis and treatment. As these arrhythmias most commonly occur within the first few days after surgery, atrial epicardial wires may be in place and can be exceedingly helpful with the diagnosis.^12,13^

The use of atrial epicardial wires for rhythm identification was first popularized by Waldo et al. in the 1970s and 1980s after demonstrating their superior ability to establish a diagnosis over the standard ECG.^14,15^ This improvement in diagnosis has been confirmed by others.^16,17^ Nevertheless, despite the American Heart Association practice standards that recommend atrial signal interpretation to improve the accuracy of diagnosis over surface ECG signals,^10^ even in patients with atrial epicardial wires, AEGs are not universally used for the diagnosis of arrhythmias.

For some practitioners, proper interpretation of the atrial signals can be challenging. Additionally, until recently, AEGs were not displayed continuously or in real time on the bedside monitor. This lack of timeliness resulted in a delayed or missed diagnosis and less utility from an AEG. Fortunately, real-time AEG monitoring displayed on the bedside monitor is now possible (AtriAmp; Atrility Medical, Madison, WI, USA) and has now been demonstrated in pediatric patients at our institution.^11^ We have found that the ability to review the AEG on the hospital telemetry system has also allowed for improvements in the diagnosis of arrhythmias that are transient. As the use of the AtriAmp becomes more common, further considerations such as atrial wire location and standard adjustments to the bedside monitor may further optimize the appearance of the AEG.

## Conclusion

Postoperative arrhythmias are common after cardiac surgery and may result in increased morbidity and mortality. Despite a more accurate rhythm identification utilizing the AEG, providers may not obtain AEG due to the lack of provider comfort with AEG interpretation. Therefore, in an attempt to improve provider comfort with the interpretation of the AEG, we herein presented an algorithm for tachyarrhythmia identification of postoperative arrhythmias using AEGs.

## Figures and Tables

**Figure 11:**
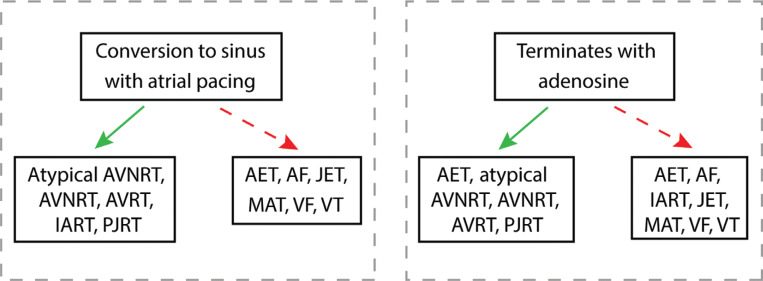
The response of arrhythmias to rapid atrial overdrive pacing and to adenosine; relevant arrhythmias are denoted with an asterisk within the preceding algorithms. It should be noted that some arrhythmias may respond variably to atrial pacing or adenosine. Therefore, the response of arrhythmias to adenosine and/or pacing may not be diagnostic but can often help with identification or treatment. AET: atrial ectopic tachycardia; AF: atrial fibrillation; AVNRT: atrioventricular nodal reentrant tachycardia; AVRT: atrioventricular reentrant tachycardia; IART: intra-atrial reentrant tachycardia; JET: junctional ectopic tachycardia; MAT: multifocal atrial tachycardia; PJRT: permanent junctional reciprocating tachycardia; VF: ventricular fibrillation; VT: ventricular tachycardia.
